# Large genomic fragment deletion and functional gene cassette knock-in via Cas9 protein mediated genome editing in one-cell rodent embryos

**DOI:** 10.1038/srep17517

**Published:** 2015-12-01

**Authors:** Liren Wang, Yanjiao Shao, Yuting Guan, Liang Li, Lijuan Wu, Fangrui Chen, Meizhen Liu, Huaqing Chen, Yanlin Ma, Xueyun Ma, Mingyao Liu, Dali Li

**Affiliations:** 1 Shanghai Key Laboratory of Regulatory Biology, Institute of Biomedical Sciences and School of Life Sciences, East China Normal University, Shanghai 200241, China; 2 The Key Laboratory of Adolescent Health Assessment and Exercise Intervention of Ministry of Education, East China Normal University, Shanghai 200241, China; 3 Hainan Provincial Key Laboratory for human reproductive medicine and Genetic Research, Hainan Reproductive Medical Center, the Affiliated Hospital of Hainan Medical University, Haikou 570102, China; 4 The Institute of Biosciences and Technology, Texas A&M University Health Science Center, Houston, Texas 77030, USA

## Abstract

The CRISPR-Cas RNA-guided system has versatile uses in many organisms and allows modification of multiple target sites simultaneously. Generating novel genetically modified mouse and rat models is one valuable application of this system. Through the injection of Cas9 protein instead of mRNA into embryos, we observed fewer off-target effects of Cas9 and increased point mutation knock-in efficiency. Large genomic DNA fragment (up to 95 kb) deletion mice were generated for *in vivo* study of lncRNAs and gene clusters. Site-specific insertion of a 2.7 kb CreERT2 cassette into the mouse *Nfatc1* locus allowed labeling and tracing of hair follicle stem cells. In addition, we combined the Cre-Loxp system with a gene-trap strategy to insert a GFP reporter in the reverse orientation into the rat *Lgr5* locus, which was later inverted by Cre-mediated recombination, yielding a conditional knockout/reporter strategy suitable for mosaic mutation analysis.

Genetically engineered animals, especially the mouse and the rat, are of great value not only for basic research but more importantly for modeling diseases and developing therapeutic strategies^1^. The establishment of site-specific genomic engineering through autonomous homologous recombination in mouse embryonic stem cells represented a major scientific breakthrough^2^,^3^. Although conventional gene targeting is costly, labor and time intensive, and inefficient^1^,^4^, it has been used for modification of large genomic DNA fragments^5^,^6^,^7^. Through generation of site-specific DNA double-strand breaks (DSBs), programmable nucleases (such as zinc finger nucleases (ZFNs), transcription activator-like effector nucleases (TALENs) and clustered regularly interspaced short palindromic repeat (CRISPR)-associated 9 (Cas9) systems) have been widely used in precise genomic editing to generate knockout, knock-in and chromosomal rearrangements in cells and animals^8^,^9^,^10^,^11^. Distinct from ZFNs and TALENs which recognize the target genomic loci via a pair of tandem repeats, the modified CRISPR/Cas system recognizes the target site through a core endonuclease, Cas9, and a synthetic single guide RNA (sgRNA) that directs Cas9 to its target DNA through Watson-Crick base-paring rules. The only requirement of the DNA target is the existence of a PAM (protospacer-adjacent motif) sequence (-NGG for Cas9 from *S. pyogenes*) directly downstream of the seed region of the target site^12^,^13^. Due to its flexibility, convenience and high efficiency, many potential applications of CRISPR/Cas system have been explored in depth^14^. However, the problem of off-target effects of Cas9 mediated gene targeting is still a great concern for researchers although a series of studies have demonstrated improved specificity through double-nicking or shortening the sgRNA length^15^,^16^.

The high frequency off-target effects in cells with the CRISPR/Cas system is hypothesized to be partially due to prolonged nuclease activity in stable cell lines^17^. Recently, two groups reported that through introduction of recombinant Cas9 protein and sgRNAs into cells, both the activity and the accuracy of the CRISPR/Cas system has been improved^17^,^18^. Although initial work suggested highly efficient generation of gene knockout zebrafish and mice through direct injection of Cas9 protein/sgRNA^19^,^20^, only a few reports described the successful knockout of a large DNA fragment^21^ or knock-in of a functional gene cassette using this method^22^,^23^,^24^. Here, we investigated the generation of multiple animal models using Cas9 recombinant protein for large genomic DNA deletion and functional gene cassette knock-in. In addition, we found that injection of Cas9 protein rather than Cas9 mRNA slightly increased the knock-in efficiency and lowered the incidence of off-target effects. Our study suggests that recombinant Cas9 protein is an excellent alternative choice for generation of various genetically modified animal models.

## Results

### Deletion of a large genomic fragment in mice by injection of Cas9 protein and sgRNA pairs

Previously, we and others successfully generated mutant mouse and rat models through injection of either DNA or RNA encoding corresponding Cas9/sgRNA^24^,^25^,^26^,^27^. As delivery of Cas9 protein is an alternative method with potential advantages that has not been fully tested in model organisms^19^, here we sought to extensively expand the applications for generation of genetically modified animals through injection of Cas9 protein. The recombinant Cas9 protein was purified and its activity was confirmed in an *in vitro* cleavage test using commercial Cas9 enzyme as a positive control. (Data not shown).

Creating small indels usually could not disrupt long non-coding RNA (lncRNA) genes due to the lack of an open reading frame^28^. Although previous reports have shown a maximum deletion of 10 kb of genomic DNA via direct injection of two sgRNAs into one cell mouse embryos^21^,^29^, a further attempt to delete a 30 kb genomic region was unsuccessful^21^. Moreover, the performance of Cas9 protein injection to generate large genomic deletions has not been reported. To test the efficiency of Cas9 protein mediated large DNA fragment deletion we injected Cas9 protein into one-cell mouse embryos together with two sgRNAs targeting exon1 and 2 of lncRNA GM14005 genomic DNA, a region spanning 53 kb (Supplementary Fig. S1a). Among 5 F0 pups, 1 founder was confirmed by PCR genotyping with subsequent sequencing (Supplementary Fig. S1b and Table 1). The 53 kb deletion was efficiently transmitted to the F1 generation (Supplementary Fig. S1b).

**Figure 1:**
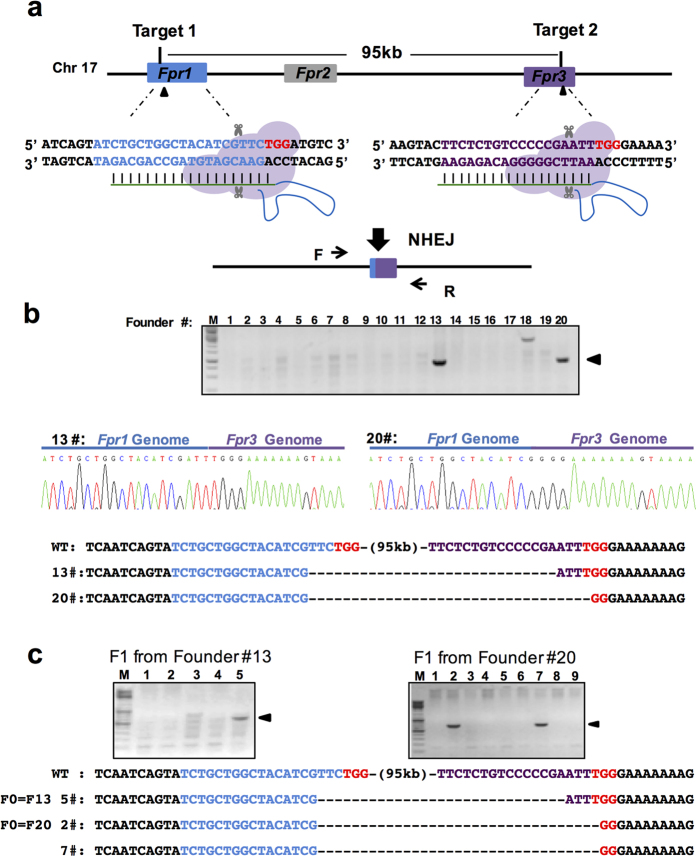
Deletion of the Fpr1-3 gene cluster by two sgRNAs spanning 95 kb. (**a**) Schematic overview of the strategy to delete a 95 kb DNA fragment on chromosome 17. The exons *Fpr1* and *Fpr3* are labeled in blue and purple respectively, and the target sites are indicated by arrowheads. PAM sequences are in red following the target sequence highlighted in blue or purple. After deletion of the DNA fragment, the resulting genomic sequence is composed of the 5′ part of Fpr1 (blue) and the 3′ portion of Fpr3 (purple). The locations of PCR primers (F, Forward; R, reverse) are indicated by arrows. (**b**) (Top) Genotyping of the founders. PCR analysis of F0 mice injected with Cas9 protein and sgRNAs listed in (**a**). Arrowheads indicate the founders with deletion of the 95 kb genomic DNA fragment. (Below) Sequencing data of the PCR products from two founders showing the joint *Fpr1/Fpr3* genomic sequence. M, DNA marker. (**c**) Genotyping analysis of F1 progenies from two founders showing germ line transmission of the 95 kb deletion. M, DNA marker.

As some homologous genes are located adjacent to one another to form a gene cluster, it is difficult to generate multiple-gene knockout animals by crossing due to the low homologous recombination efficiency within short distances in the genome. Therefore, we next tried to delete a genomic region which spans 95 kb in the mouse genome containing three receptors for formyl peptide to extend the deletion range through direct embryo injection (Fig. 1a). After injection of Cas9 protein:sgRNAs, 20 F0 pups were obtained. PCR and subsequent DNA sequencing confirmed that two pups were founders bearing the desired 95 kb genomic DNA deletion (Fig. 1b, Table 1). The deletion was germline transmissible as determined by genotyping of F1 progeny (Fig. 1c). We demonstrated that through a single injection of Cas9 proteins and two sgRNAs, large DNA fragment deleted animals could be easily generated.

### Off-target effect studies of Cas9 protein-mediated genome editing in mouse embryos

**Figure 2:**
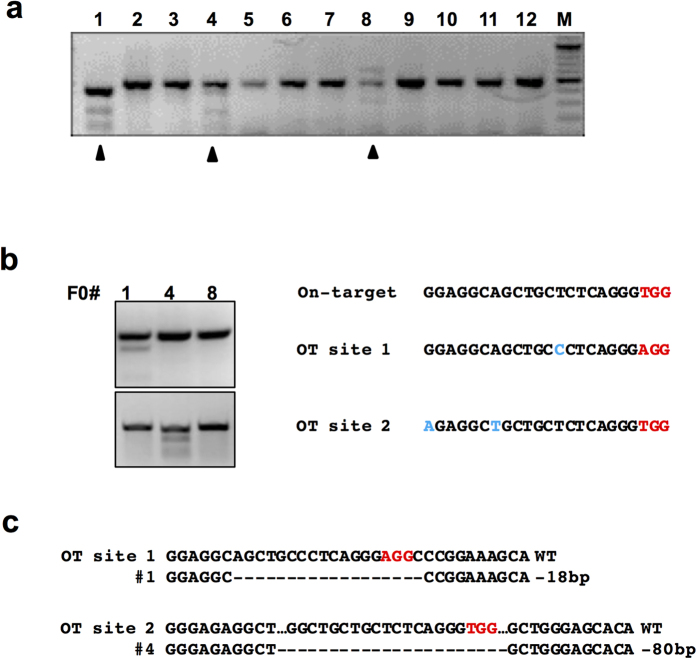
Off-target analysis of sgRNA targeting the AR genomic locus. (**a)** Genomic DNA extracted from 12 F0 pups was subjected to PCR with on-target primers and subsequent T7E1 digestion. On-target mutants are indicated by arrowheads. M, DNA marker. (**b**) T7E1 digestion analysis of founders on two off-target (OT) sites. The sequence of On-target and OT sites are listed. The PAM sequences are in red and mismatched nucleotides are in blue. (**c**) The sequences of the OT sites of the founders bearing mutations are listed.

It was reported that reducing the amount of Cas9/sgRNA plasmid DNA during transfection increased the cleavage specificity^30^. As Cas9 protein exhibits a shorter duration than DNA/RNA transfected into the cell, we assumed that injection of Cas9 protein could be helpful to increase the targeting specificity. To check for potential off target activities by Cas9 protein, we chose a previously reported sgRNA that targets the mouse androgen receptor (*Ar*) locus^31^. After injection of Cas9 protein:sgRNA, 3 on-target mutant F0 pups were identified by T7E1 digestion (Fig. 2a). The efficiency was comparable with a previous report^31^. Two potential off-target sites were analyzed by T7E1 assay followed by sequencing (Fig. 2b,c). For each off-target site, 1 of 3 founders exhibited off-target digestion (Fig. 2b,c). It was reported that all 3 on-target founders contained both of these two off-target mutations in mice generated via injection of Cas9 mRNA^31^. The off-target frequency was reduced when we injected Cas9 protein, but the on target efficiency was consistent with previous reports^17^. This is probably due to the shorter half-life of Cas9 protein compared to Cas9 mRNA and its subsequent translation^17^,^32^. The results suggested that Cas9 protein injection could be used as an alternative way to generate mutations with the potential benefit of reducing off-target digestion.

### Comparing the HDR efficiency mediated by Cas9 protein and Cas9 mRNA

Nuclease-induced DNA double-strand breakage stimulates HDR efficiency. To test the efficiency of Cas9 protein-mediated point mutations, we tried to generate an Arg84Thr (ga > cg) mutation in the mouse *Sin1* genomic locus (Supplementary Fig. S2a). As the mutation site is outside the sgRNA seed region, we designed the donor template by introducing 6 synonymous mutations within the sgRNA target sequence to avoid re-digestion of the target after HDR. In addition, to test whether a mutation outside the targeting sequence can be introduced into the genome, we also designed a substitution 35bp away from the PAM to introduce a restriction enzyme (PvuII) recognition site (Supplementary Fig. S2a). After pronuclear injection, 11 F0 pups were obtained and 9 of them contained a modified *Sin1* locus (Supplementary Fig. S2b, Table 1). Although 3 pups (#5, #8 and #11) received the 6 synonymous mutations, the desired Arg84Thr (ga > cg) mutation was only identified in 1 pup (#8). In F0 pup #5, a 61 bp random sequence was inserted 5′ to the target sequence. In F0 pup #11, the mutant sequence was inserted near the PAM. Although nucleotide substitutions occurred in 7 of 9 F0 pups, none of them carried the g > c mutation 35 bp away from PAM, suggesting that mutation far away from the PAM is difficult to introduce.

**Figure 3:**
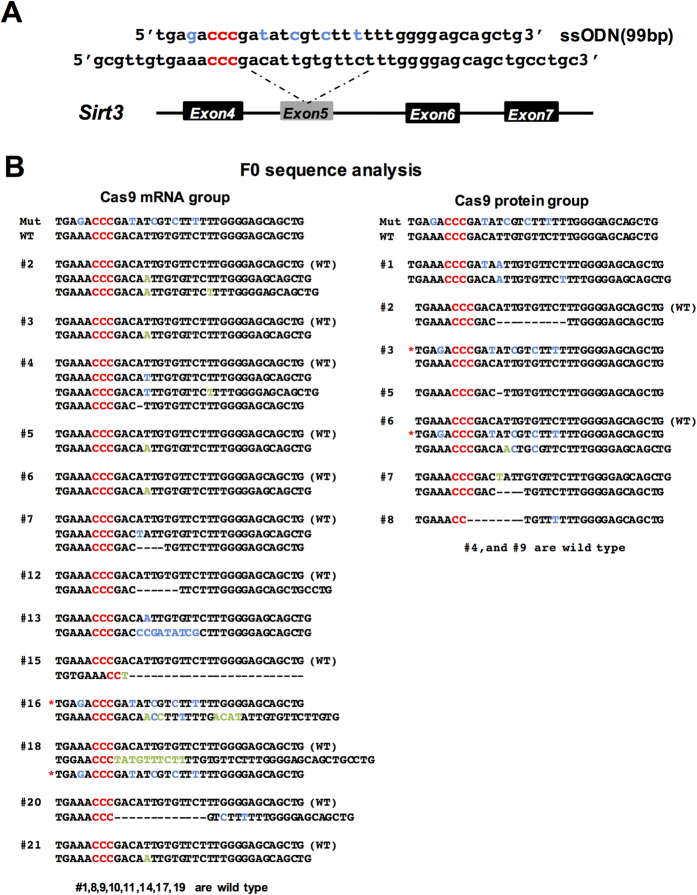
Comparison of the knock-in efficiency between injection of Cas9 protein and Cas9 mRNA. (**a**) Schematic overview of the strategy to introduce specific mutations in the Sirt3 locus. PAM sequence is labeled in red, designed mutations are blue. (**b**) The sequence of F0 pups generated from Cas9 mRNA (left) or protein (right) injection are listed. Founders with the desired mutation are labeled with a red asterisk. PAM sequence is red, designed mutations are blue, random mutations are green.

Our above results suggest that delivery of Cas9 protein:sgRNA:ssODN is capable of introducing mutations through an HDR mechanism. Since HDR is cell cycle dependent and Cas9 protein should function faster than Cas9 mRNA once injected into the pronucleus, we assumed that the HDR efficiency following Cas9 protein injection was different from that of Cas9 mRNA injection. To compare the HDR efficiency between injection of Cas9 protein and Cas9 mRNA, we designed an sgRNA targeting the mouse *Sirt3* locus and synthesized the corresponding ssODN template which contains 4 substitutions in the target sequence and 1 substitution near the PAM on the opposite side (Fig. 3a). Of 21 newborn pups in the Cas9 mRNA group, 12 pups carried indels. Only 2 of them were founders bearing the desired knock-in mutation. In the Cas9 protein injection group 7 of 9 pups carried indels with 2 of them bearing the desired knock-in mutation. These results suggested that injection of Cas9 protein could give a slightly higher indel (7/9 = 78% vs 12/21 = 63%) and increased HDR (2/9 = 22% vs 2/21 = 9.5%) rate than Cas9 mRNA injection (Fig. 3b and Table 1); however more experiments should be carried out to confirm this phenomenon on different genomic loci. In addition, the group that received Cas9 mRNA injection exhibited higher chimerism in some pups compared to the Cas9 protein injection group (Fig. 3b).

### Generation of Nfatc1-IRES-CreERT2 knock-in mice for lineage tracing

**Figure 4:**
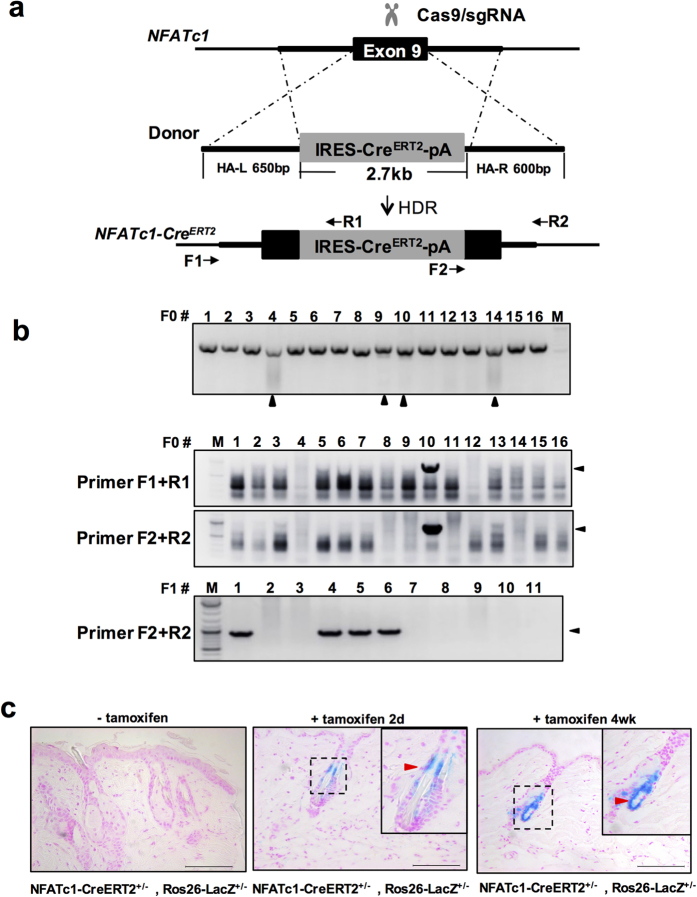
Site-specific insertion of IRES-Cre^ERT2^-pA into the mouse *Nfatc1 locus*. (**a**) Schematic of the strategy for insertion of the CreERT2 cassette into the mouse *Nfatc1* locus. Cas9/sgRNA targeted the 3′UTR of the *Nfatc1* gene in Exon 9. The HDR donor sequence consists of IRES-CreERT2-polyA (2.7 kb) flanked by two homologous arms 650 bp (left-arm) and 600 bp (right-arm) in length. Positions of genotyping primers are indicated by arrows. (**b**) Genotyping of Nfatc1-CreERT2 mice. Upper, T7E1 assay to check the indels created by CRISPR/Cas. Middle, identification of founder (F0) and F1 Nfatc1-CreERT2 mice by primer pairs: F1 + R1 and F2 + R2. Arrowheads: desired bands of site-specific inserted pups. M, DNA marker. (**c**) Lineage tracing of skin Nfatc1 stem cells in Nfatc1-CreERT2^+/−^:Rosa26-LacZ^+/−^ mouse via X-gal staining. Lineage tracing began at the age of 4 weeks by intraperitoneal injection of tamoxifen. Mice dorsal skin tissue was stained 2 days (middle) or 4 weeks (right) after induction. Scale bar, 100 μm.

Site-specific insertion of functional cassettes containing relatively long DNA fragments into the mouse or rat genome through customized nucleases is challenging. As the CreERT2 cassette is widely used for inducible knockout and lineage tracing studies, we next tried to insert a 2.7 kb IRES-CreERT2 cassette into the mouse *Nfatc1* locus (Fig. 4a). We constructed a donor template with about 600 bp homology arms flanking the IRES-CreERT2 cassette. Among 16 F0 pups 4 carried indels as shown by T7E1 assay (Fig. 4b upper) and 1 founder was identified with two sets of genotyping primers (Fig. 4b, Table 1). The insertion was also confirmed by sequencing the PCR products (data not shown). We observed highly efficient germline transmission in the F1 generation (Fig. 4b). To test if the inserted CreERT2 cassette functions *in vivo*, the *Nfatc1-CreERT2* strain was crossed with the *Rosa26-LSL-LacZ* reporter strain. As *Nfatc1* is expressed in CD34^+^ hair follicle stem cells^33^, we initiated cell lineage tracing by intraperitoneal injection of tamoxifen at the age of 4 weeks when the hair cycle is in the transition from first telogen to second anagen. 2 days after induction, LacZ positive cells were detected in the bulge area of the hair follicles (Fig. 4c mid), suggesting that Nfatc1 marks the bulge cells during anagen as reported^33^. 4 weeks after induction when the hair follicles were in the second telogen stage, LacZ positive cells occupied the entire hair follicle (Fig. 4c right), suggesting that Nfatc1-positive cells are follicle stem cells in the bulge. When induced at the 8^th^ week for 2 days, only stem cells in the bulge area exhibited β-Gal staining (data not shown). To our knowledge, this is the first evidence to show that Nfatc1 marks the hair follicle stem cells through lineage-tracing studies.

### Cas9 protein mediated direct insertion of a DNA cassette into the *Lgr5* locus to generate a conditional knockout rat allele with a reporter

**Figure 5:**
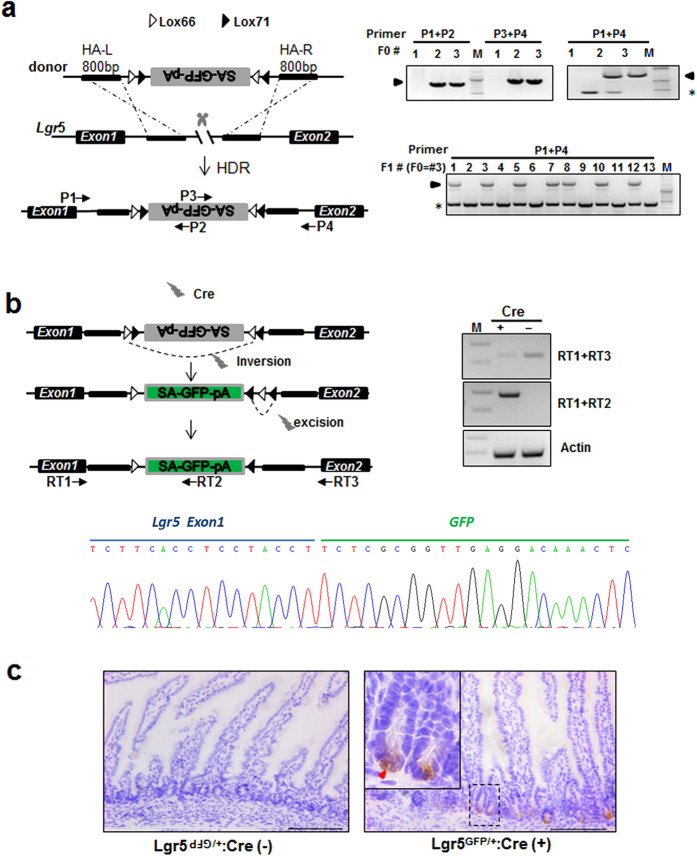
Site-specific insertion of reverse oriented SA-GFP-pA into the rat *Lgr5 locus*. (**a**) Left, schematic of the strategy to insert the inverted GFP reporter cassette into intron1 of the rat *lgr5* locus. The reporter cassette consists of a splice acceptor (SA), GFP and polyA (pA) sequences flanked by heterospecific Loxp twin-sites in opposite orientations and homologous arms each about 800 bp long. Right, Genotyping of the founder and the F1 heterozygous mice. Arrowheads indicate the detection of the GFP reporter cassette insertion into the *Lgr5* locus in F0 or F1 rats. Asterisk, the PCR band amplified from the wild-type allele. Arrows represent the positions of genotyping primers. M, DNA Marker. (**b**) Left, schematic depiction of Cre mediated cassette inversion, Loxp site excision and *lgr5* inactivation. The reporter cassette was first inverted by Cre recombinase between either inverted Loxp pairs. Thereafter, Cre mediated excision deletes the sequence in between the identically-oriented Loxp pairs, leaving one WT and one mutant Loxp site. Right, RT-PCR to detect endogenous *Lgr5* mRNA by primer pair RT1&RT3 and Lgr5-GFP fusion mRNA by primer pair RT1&RT2. Arrows represent the positions of RT-PCR primers. Lower, sequencing result indicating the fusion of Lgr5 and SA-GFP-pA. M, DNA Marker. (**c**) Detection of GFP (red arrowhead) in rat small intestine tissue after Cre-mediated recombination through immunohistochemical staining. Scale bar, 100 μm.

Mosaic mutant analysis is a powerful strategy to study individual mutant cells in a wild-type cellular environment. The challenge of this technique is to label the truly knockout cells while avoiding mis-labeling wild-type cells when the reporter activation is independent of conditional knockout of the target gene^34^. We attempted to generate a conditional knockout rat strain whose cells can be labeled after disruption of the target gene by Cre recombinase. We took advantage of the gene trap strategy together with Cre-Loxp-mediated DNA inversion^35^ to inactivate the target gene while simultaneously labeling cells with GFP (Fig. 5a,b). Lgr5 is a biomarker of adult stem cells in certain tissues, including the intestine, stomach, hair follicle^36^. However, the study of adult stem cells is hampered in rats due to the lack of reporter strains. We intended to generate an Lgr5 conditional knockout/reporter line by using the above strategy to facilitate monitoring adult stem cells in their native context. Using the Cas9 protein/sgRNA system, we inserted a DNA cassette (composed of a splice acceptor (SA) sequence, the green fluorescent protein (GFP) coding sequence and a polyadenylation (PA) sequence flanked by two pairs of lox66 and lox71 sites) in the reverse orientation into intron 1 of the rat *Lgr5* locus (Fig. 5a left). After PCR and subsequent sequencing, 2 founders were confirmed from 3 F0 pups (Fig. 5a right, Table 1). Interestingly, founder #3 was highly likely to carry homozygous mutation identified from tail DNA. However, after crossing with a wild-type rat, only half of the F1 generation from founder #3 was heterozygous suggesting that the founder was a mosaic in germ cells (Fig. 5a right). In principle, upon Cre-mediated recombination, the reverse-oriented SA-GFP-pA cassette will be inverted, then produce a truncated Lgr5 protein fused with GFP. Thus the GFP fusion proteins will label the cells having a spontaneous disruption of the endogenous *Lgr5* gene (Fig. 5b left). To test whether the cassette is functional *in vivo*, we injected Cre mRNA into heterozygous one-cell embryos due to the unavailability of the appropriate rat Cre line. As shown in Fig. 5b, the reporter cassette was inverted in the genome, and the *Lgr5-GFP* fusion mRNA was detected in the rat intestine and confirmed by sequencing. Furthermore, the GFP^+^ cells were detected in the bottom of the intestinal crypts where Lgr5^+^ stem cells reside (Fig. 5c and Supplementary Fig. S3). To our knowledge, this is the first report of marking stem cells in the rat intestine. Intercrossing F1 heterozygotes of Lgr5^gfp/+^ rats yielded 35 F2 progenies including 13 wild types and 22 heterozygotes (WT: HZ≈1:1.7 ). The lack of homozygous rats in the F2 progenies indicates prenatal lethality of *Lgr5* knockout in rats which is a more severe phenotype than that observed in *Lgr5* knockout mice which exhibited neonatal lethality^37^.

## Discussion

The CRISPR/Cas system is a powerful tool for genetic modification in cells and animals. Although multiple applications such as generation of indels, deletions, Loxp-inserted conditional alleles, and insertion of GFP reporters have been reported through genomic engineering in mammalian model organisms^24^,^27^, the execution of complicated genomic editing via Cas9 in embryos has not been extensively studied. In this report, through injection of Cas9 protein, we generated mouse strains with large genomic DNA deletions as well as mouse or rat strains with site-specific insertion of Cre or reporter cassettes. Our study extensively confirmed that injection of recombinant Cas9 protein is an alternative strategy to generate a variety of genetically modified mammalian models.

Through large DNA fragment deletion by Cas9/sgRNA, it is practicable to generate mouse strains to model human genetic disorders which are caused by the loss of large DNA fragments. Although a recent publication reported the generation of mice with a 1.6 Mb deletion in their genome, technically the authors used ESC aggregation to generate the mouse after screening for the right ESC clone^38^. This procedure takes at least 10 weeks to get the F0 chimera whose germline transmission efficiency varies case by case. Generation of genetically modified animals with Cas9 through an ESC-based strategy is still an expensive, time consuming and laborious strategy. Recent publications have shown generation of a 10 kb deletion of mouse genomic DNA by direct injection of Cas9 mRNA/sgRNA^21^,^29^. Our current work expanded the upper limit of large-scale genomic DNA deletion by creating F0 founder mice with LncRNA GM14005 genomic DNA (53 kb) deletion or formyl peptide receptor paralog (Fpr1-3) gene cluster (95 kb) deletion at high efficiency within 4 weeks. To our knowledge, this is the largest DNA fragment deletion in mammalian models generated through gene editing technology in embryos suggesting the potential of CRISPR/Cas in the study of polygenic diseases caused by contiguous genes.

Double-nicking Cas9 and dCas9-FokI strategies were effective in greatly reducing the incidence of off-target digestion^31^,^39^. However, these two strategies decrease the on-target activity of Cas9 nuclease. It was reported that introduction of Cas9 protein:sgRNA into cells decreased off-target effects but not on-target digestion^17^,^19^. Our results also suggested that it is feasible to reduce off-target efficiency through injection of Cas9 protein in embryos. This is likely because the Cas9 protein has a relatively shorter half-life in embryos than Cas9 mRNA, yielding fewer off-target hits. Site-specific knock-in or substitution is valuable for gene editing; however the efficiency is pretty low due to inefficient homologous recombination. Three targets were injected both through mRNA and protein in parallel experiments and the frequency of chimerism was decreased in the protein injected groups (Fig. 3 and data not shown). In our experience, the HDR efficiency varies from one genomic locus to another. In some cases, the sgRNA efficiency to induce NHEJ mutations is high (above 60%), but yields no knock-ins after several rounds of injection (data not shown). It is not clear whether it depends on the chromatin status or not. We used the *Sirt3* locus to compare the knock-in efficiency of Cas9 mRNA injection and protein injection. Our data suggested that the HDR rate is slightly increased in the protein injected group (Fig. 3b), which is consistent with the results of cell cycle dependent introduction of Cas9 protein into cell lines^32^. More experiments need to be done to further confirm that the HDR efficiency is increased through Cas9 protein injection. A recent study is consistent with our results that injection of Cas9 protein in mouse embryos led to higher HDR efficiency, although the authors used dual-crRNA:tracrRNA instead of sgRNA^22^.

Knock-in of large DNA fragments into embryos is challenging for gene editing technology due to low efficiency. We showed that a functional 2.7 kb CreERT2 cassette was successfully inserted into the mouse *Nfatc1* locus. As the coding sequence of more than 85% of human protein-coding genes is under 3 kb^40^, in principle the CRISPR/Cas system can be used to introduce most human genes for the generation of humanized models. We also developed a strategy for mosaic mutant analysis which can be used to label the cells once the target gene is inactivated by Cre recombinase (Fig. 4d,e). In our Lgr5 conditional knockout/reporter stain, the GFP fluorescence could not be directly detected through microscopy. It is possible that the *Lgr5* promoter is not strongly expressed in the rat intestine. It is also possible that the N-terminus of Lgr5 fused to GFP after cassette inversion disrupted the structure necessary for GFP fluorescence. Our results suggest it is better to avoid the production of fusion proteins by insertion of IRES or T2A sequences between the SA and the GFP sequence. Although the strategy worked well in this case, there are two concerns remaining. First, it is not clear if the inverted insertion of the L-SA-GFP-PA-L cassette in the intron affects the endogenous gene. Second, after Cre-mediated recombination, the cassette may not completely inactivate the endogenous gene as sometimes the artificial exon is neglected as reported in some cases of gene-trapped mouse models. We also noticed a lower efficiency in the *Nfatc1* locus knock-in experiment than in the *Lgr5* locus. This is probably due to the longer knock-in cassette (Nfatc1: 2.7 Kb vs Lgr5: 1.1 Kb) and lower sgRNA activity (Nfatc1, 25% vs Lgr5, 66%). Although other group suggested that higher HDR efficiency was achieved in some genomic loci in rat embryos^41^,^42^, it is difficult to evaluate the HDR efficiency in *Lgr5* locus due to limited pups obtained in this study.

In summary, this report suggests that the performance of Cas9 protein is comparable or even better than Cas9 mRNA for generation of genetically modified animal models, although more experiments should be done to confirm this. Our results exhibit the great potential of Cas9 in creating genetically modified mice and rats, paving its way for future applications in disease modeling for basic scientific research and in humanized models for gene therapies.

## Materials and Methods

### Recombinant Cas9 expression and purification

The Cas9 open reading frame was amplified from pX330^43^ (Addgene #42230) and cloned into pET28a in frame with an N-terminal His-6 tag for *in vitro* expression. Cas9 protein was expressed in Transetta (DE3) (Transgen Biotech), induced at OD 0.8 with 0.2 mM IPTG followed by at least 16 hours of expression at 18 °C. The protein was bound to Ni-NTA Agarose (Qiagen) and washed extensively with 20 mM Tris PH 8.0, 500 mM NaCl, 10% glycerol, followed by linear gradient elution with 500 mM imidazole, 20 mM Tris PH 8.0, 250 mM NaCl, 10% glycerol. Fractions over 90% purity were dialyzed against 150 mM KCl, 20 mM Tris PH 7.5, 1 mM DTT, 10% glycerol, aliquoted, and stored at −80 °C.

### *In vitro* sgRNA and Cas9 mRNA synthesis

DNA templates of Cas9 and sgRNAs were first amplified by PCR using 5′ primers containing SP6 and T7 promoters respectively as previously described^21^. *In vitro* transcription of Cas9 mRNA was performed using the mMESSAGE mMACHINE kit (Ambion). sgRNAs were transcribed *in vitro* by T7 transcription kit (Takara) according to the manufacturer’s instructions. All single stranded oligonucleotide DNA donors were ordered from Biosune Inc.

### Microinjection of zygotes

All animal experiments were performed in accordance with the regulations of the Association for Assessment and Accreditation of Laboratory Animal Care in Shanghai and were approved by the East China Normal University Center for Animal Research (AR2013/04009).

Microinjection was performed as described previously^22^. C57BL/6J mice and Sprague Dawley (SD) rats were purchased from SLAC Shanghai. In brief, mouse and rat one-cell embryos were obtained by superovulation of females mated with males with the same genetic background. The embryos were harvested in M2 medium and cultured in KSOM medium (Millipore) for 2–3 hours. In the knock-in experiments, TE solution containing 12.5 ng/ul sgRNA, 10–50 ng/ul DNA donor, 30 nM Cas9 protein (or 25 ng/μl Cas9 mRNA in the Sirt3 point mutation experiment) was injected into the pronuclei of one-cell stage embryos. In the *Ar* knockout experiment 30 nM Cas9 protein was injected into the cytoplasm of the one-cell stage embryos with the corresponding sgRNA. Injected embryos were transferred into pseudopregnant female mice or rats immediately after injection or the next morning after overnight culture in KSOM.

### Genotyping and sequence analysis

Genomic DNA from tails of the progenies was extracted and subjected to PCR using the indicated primers followed by DNA sequencing analysis. To check for point mutations and off-target effects, the genomic region encompassing the targeted region was PCR amplified, purified and reannealed followed by digestion with T7 endonuclease I (NEB or Beijing Polymath) and then analyzed by agarose gel electrophoresis. The exact sequences were determined by Sanger sequencing. Sequences of all primers are listed in Table S1.

### β-Galactosidase Staining

Nfatc1-CreERT2:Rosa26-LSL-lacZ mice were injected intraperitoneally with 100 μl tamoxifen (20 mg/ml) at the age of 4 weeks. Mice were euthanized 2 days or 4 weeks after tracing and mouse dorsal skin tissue from the mid back was collected, washed with ice-cold LacZ fixing buffer (2% formaldehyde, 0.2% glutaraldehyde, 0.02% Nonidet P-40), followed by incubation in fixing buffer for 2 h on a shaker at 4 °C. Then the skin tissues were washed with LacZ wash buffer (2 mM MgCl_2_, 0.01% deoxycholate, 0.02% Nonidet P-40 in PBS, PH 8.0) twice (10 min each time), followed by incubating overnight in staining buffer (0.5 mg/ml X-gal dissolved in LacZ wash buffer) at room temperature. Tissues were then fixed, embedded in paraffin and sectioned for histological analysis.

### Immunohistochemistry and immunofluorescence staining

Mice or rats were sacrificed by carbon dioxide asphyxiation. For immunohistochemistry, tissues were fixed with 4% paraformaldehyde (PFA), embedded in paraffin, sectioned at 4 μm followed by de-waxing and rehydration. Sections were incubated with 3% H2O2 in methanol for 20 min, washed in PBS, followed by antigen retrieval in 10 mM sodium citrate, PH 6.0 at 100 °C. After blocking in 1% BSA for 30 min at room temperature, rat liver sections were incubated with 1:500 diluted anti-Fah (purified from immunized rabbit serum) antibody and small intestine sections were incubated with 1:500 diluted anti-GFP antibody (Santa Cruz SC-9996) overnight. Sections were developed with diaminobenzidine and counterstained with hematoxylin following a 30 min incubation with secondary antibody. For immunofluorescence, rat small intestines were embedded in OCT, frozen, cryo-sectioned and fixed in 4% PFA. Thereafter sections were stained with anti GFP antibody and subjected to immunofluorescence microscopy.

## Additional Information

**How to cite this article**: Wang, L. *et al.* Large genomic fragment deletion and functional gene cassette knock-in via Cas9 protein mediated genome editing in one-cell rodent embryos. *Sci. Rep.* **5**, 17517; doi: 10.1038/srep17517 (2015).

## Supplementary Material

Supplementary Information

## Figures and Tables

**Table 1:** Cas9 protein:sgRNA mediated gene modification in mice and rats.

Gene	Injected embryos	Transferred embryos (%)	Live Newborns (%)	Indels/deletions (%)	HDR (%)
*Sin1*	154	83(54)	11(13)	9(82)	1(11)
*Sirt3*(mRNA)	204	120(59)	21(17)	12(57)	2(17)
*Sirt3*(protein)	144	90(62)	9(10)	7(78)	2(28)
*Gm14005*	61	51(84)	5(10)	1(20)	N/A
*Fpr1-3*	115	82(71)	20(24)	2(10)	N/A
*Nfatc1*	97	66(68)	16(24)	4(25)	1(6)
*Lgr5*	123	89(72)	3(3)	N/A	2(66)

Percentages were calculated using the number in each column as the numerator and the number in the column to its left as the denominator.
