# Supplementary material for: Large genomic fragment deletion and functional gene cassette knock-in via Cas9 protein mediated genome editing in one-cell rodent embryos
Source: Sci Rep. 2015 Dec 1;5:17517. doi: 10.1038/srep17517 (PMC4664917; doi:10.1038/srep17517)

Supplementary Information

Large genomic fragment deletion and functional gene cassette knock-in via Cas9 protein mediated genome editing in one-cell rodent embryos

Liren Wang[1,2†], Yanjiao Shao[1,†], Yuting Guan[1,†], Liang Li[1], Lijuan Wu[1], Fangrui Chen[1], Meizhen Liu[1], Huaqing Chen[1], Yanlin Ma[3], Xueyun Ma[1], Mingyao Liu[1, 4,*] and Dali Li[1,*]

[1]Shanghai Key Laboratory of Regulatory Biology, Institute of Biomedical Sciences and School of Life Sciences, East China Normal University, Shanghai 200241, China

[2]The Key Laboratory of Adolescent Health Assessment and Exercise Intervention of Ministry of Education, East China Normal University, Shanghai 200241, China.

[3]Hainan Provincial Key Laboratory for human reproductive medicine and Genetic Research, Hainan Reproductive Medical Center, the Affiliated Hospital of Hainan Medical University, Haikou 570102, China

[4]The Institute of Biosciences and Technology, Texas A&M University Health Science Center, Houston, Texas 77030, USA

* Correspondence should be addressed to: Dr. Dali Li and Mingyao Liu, Shanghai Key Laboratory of Regulatory Biology, Institute of Biomedical Sciences and School of Life Sciences, East China Normal University, Shanghai 200241, China; E-mail: dlli@bio.ecnu.edu.cn or mlliu@bio.ecnu.edu.cn

Table of Contents

Supplementary Figure 1. Generation of mouse strain with 53 kb deletion of lncRNA Gm14005 genomic DNA.

Supplementary Figure 2. Generation of site-specific mutations via Cas9 protein using ssODNs as donor template.

Supplementary Figure 3. Detection of GFP expression in Lgr5GFP/+ rat strain.

Supplementary Table 1. Numbers and percentage of injected and transferred embryos and the Indel and HDR rate

Figure S1. **Generation of a mouse strain with a 53 kb deletion of lncRNA Gm14005 genomic DNA.** (a) Schematic overview of the knockout strategy. The sequences of sgRNAs targeting exon1 and exon2 of Gm14005 are listed in blue. The PAM sequences are in red. After deletion, the positions of primers are shown with arrows. F, sense genotyping primer; R, antisense genotyping primer. (b) PCR analysis of F0 founders (left) and F1 progenies of the founder (right). Arrowhead indicates detection of successful deletion of the 53kb genomic DNA fragment. M, DNA marker.

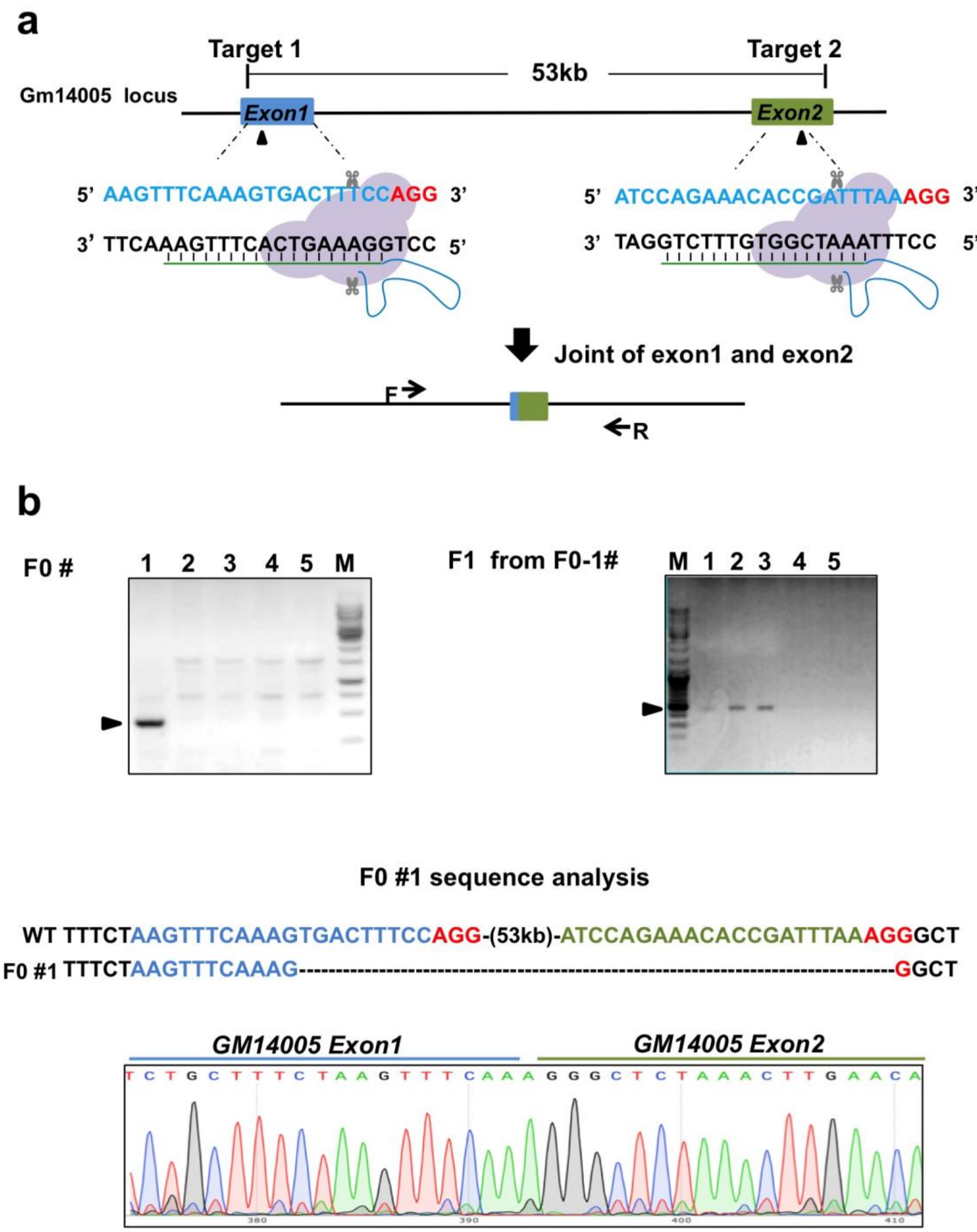

Figure S2. Generation of site-specific mutations via Cas9 protein using ssODNs as a donor template. (a) Schematic overview of the strategy to introduce specific mutations in the *Sin1* locus. PAM sequence is in red. The synonymous mutations are labeled in blue, point mutations in purple. The sgRNA targeting sequence is underlined. (b) The sequences of F0 pups are listed. Labeling is as above, with random insertions in green. The founder with the desired mutations is labeled with a red asterisk.

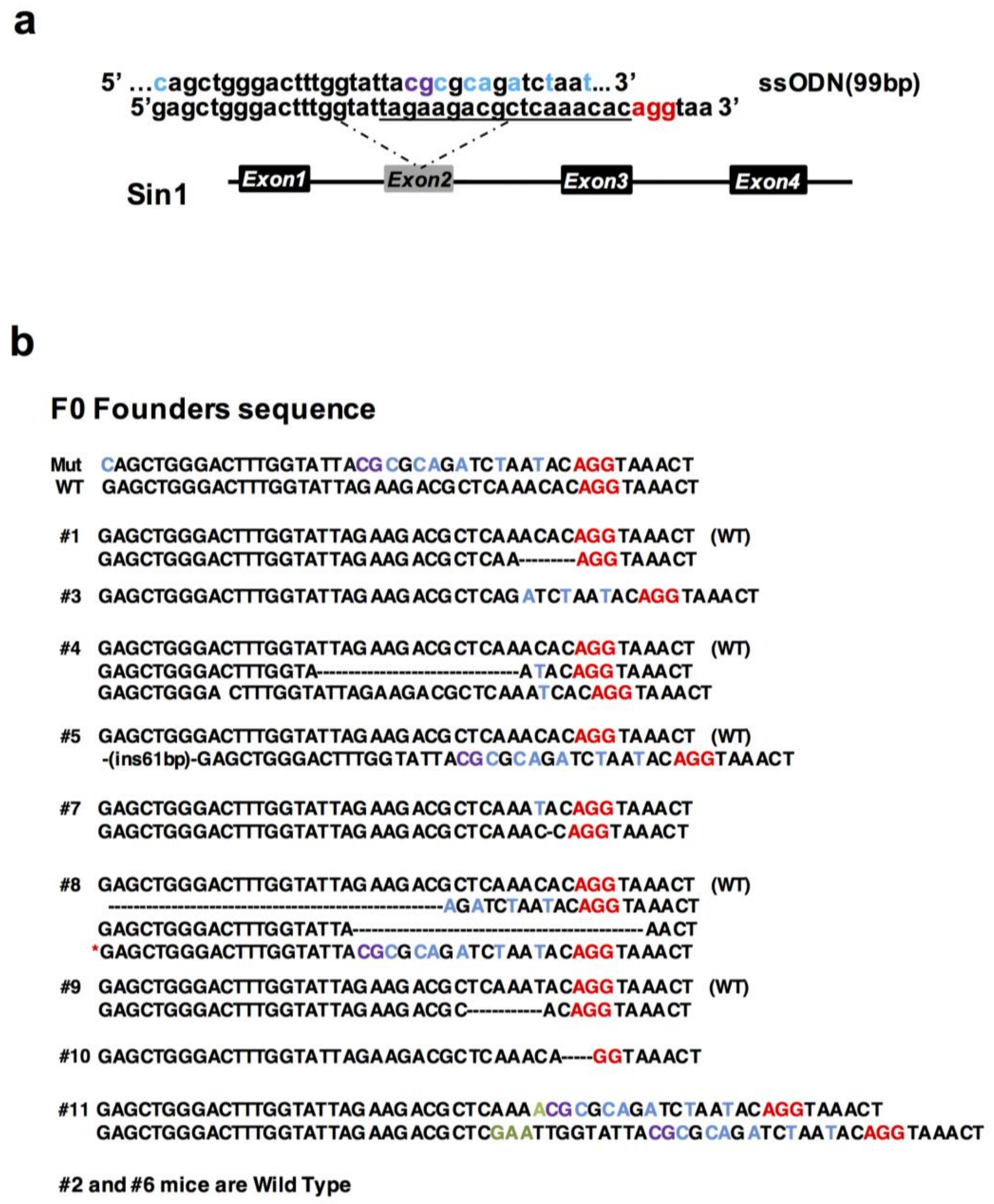

Figure S3. Detection of GFP expression in Lgr5$^{GFP/+}$ rat strain. (a) Immunofluorescent staining of the GFP protein with anti-GFP antibody in rat small intestine. Arrowheads: GFP-positive cells. Scale bar, 100μm.

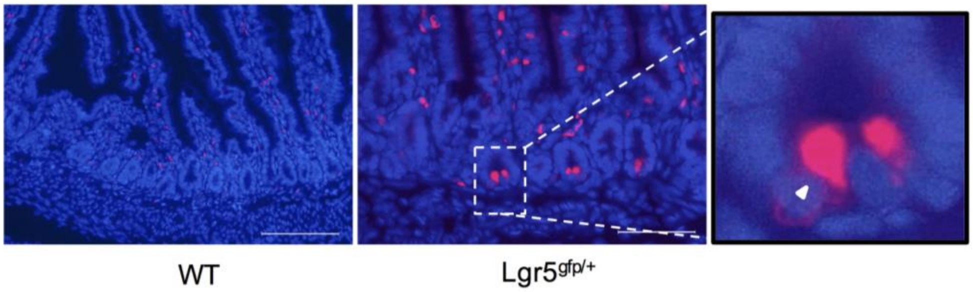

Table S1: oligonucleotides and sgRNA targeting sequences.

Genotyping primers	Sense 5'-3'	Antisense 5'-3'
Sin1	GGTGAGCCTGTGAAACAGTATG	GGCTAGCCTGCTCCTATAGTAA
Sirt3	GGGACCATTACAGAGTGAAGA	CATACAGAGCCACAGACATACC
***Fpr1-3* deletion**	TTAGGTGGAGTTATGGTAGTGG	TTCAGGTCCCAAAGACAAAC
***Nfatc1-Cre*^{ERT2} *-1* (F1, R1)**	CCTCCGGCCAATTCACAATC	ACACCGGCCTTATTCCAAGC
***Nfatc1-Cre*^{ERT}*-2* (F2, R2)**	TTTCCCTGCCACAGCTTGA	ATTTCCCGAAAGGAAGAGGC
***Lgr5-fl-gfp1* (P1, P2)**	GCCCAATATGTATGTAACAGAC	GTCCAAACTCATCAATGTATCT
***Lgr5-fl-gfp2* (P3, P4)**	TCGCCCTTGCTCACCATACT	CACGCACAGGTCATAACCAGAC
***Lgr5-fl-gfp3* (RT1, RT2)**	CTCAGCGTCTTCACCTCCTACC	GCGTTGTCATCTAGCCACAGG
Lgr5-fl-gfp4 (RT3)		GCTCGATGCGGTTCACCAG
lncRNA GM14005	CTAACCAAAGCGTCTCCTCCC	TGATGAGATTCCAGCTCCACA
**Off target primer 5'-3'**	Sense 5'-3'	Antisense 5'-3'
Ar off target 1	ATGGCTGGATATGTTTGAGA	TACATCACAAGGAAGACCAA
Ar off target 2	CCTACATAGTGATTGCCTAAC	CTTCTACACAACTGCCATC
**sgRNA Target sequence 5'-3'**		
sgSin1	ATTAGAAGACGCTCAAACAC	
sgSirt3	TCCCAAAGAACACAATGTC	
sgFpr1	CTGCTGGCTACATCGTTC	
sgFpr3	TTCTCTGTCCCCCGAATT	
sgNfatc1	TGACGTGAGACTTTAATA	
sgAr	GGAGGCAGCTGCTCTCAGGG	
sgGM14005 exon1	AAGTTTCAAAGTGACTTTCCAGG	
sgGM14005 exon2	ATCCAGAAACACCGATTTAAAGG	

Original gel images (a) Original gel image of T7E1 assay for Nfatc1 F0 identification in Fig4 b. (b) Original gel image of beta actin band in the RT-PCR assay in Fig5 b right. (c) Original gel image of *Lgr5* band in the RT-PCR assay in Fig5 b right. (d) Original gel image of *Lgr5* allele knock-in genotyping in Fig5 a right.

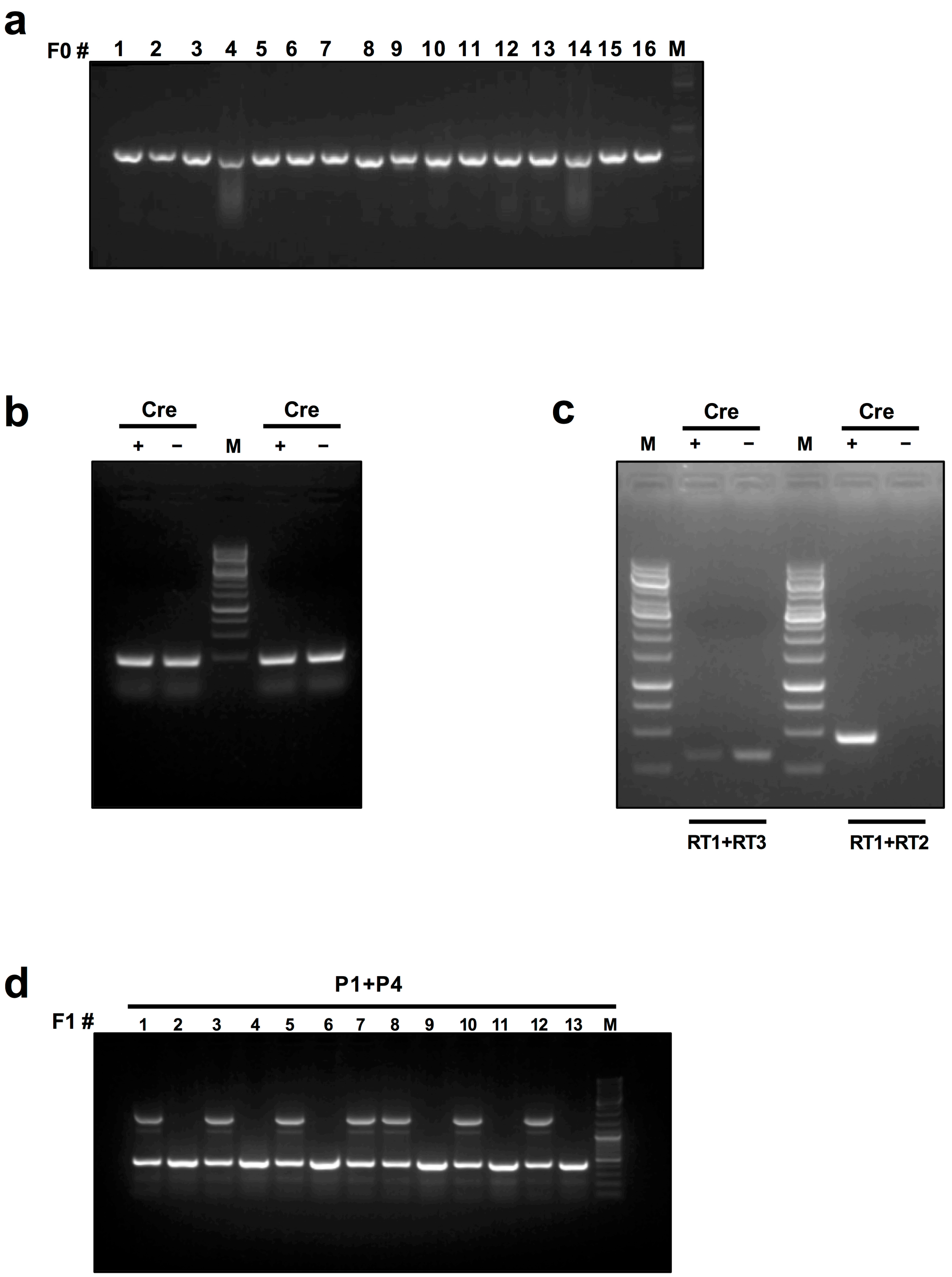

Supplement: Supplementary Information [file srep17517-s1.pdf]
